# Awareness of the Maternal Health Benefits of Lactation Among U.S. Pregnant Individuals

**DOI:** 10.1016/j.whi.2023.12.004

**Published:** 2024-02-01

**Authors:** Caidon Iwuagwu, Melissa J. Chen, Adrienne E. Hoyt-Austin, Laura Kair, Margaret Fix, Eleanor Bimla Schwarz

**Affiliations:** a Center for Healthcare Research and Policy, University of California, Davis, Sacramento, California; b Department of Obstetrics and Gynecology, University of California, Davis, Sacramento, California; c Department of Pediatrics, University of California, Davis, Sacramento, California; d Department of Medicine, University of California, San Francisco, San Francisco, California

## Abstract

**Introduction:**

We assessed awareness of the maternal health benefits of lactation among a sample of nulliparous pregnant individuals in the United States, identified variables associated with awareness of these benefits, and examined whether awareness of these benefits impacts breastfeeding attitudes or intentions.

**Methods:**

We administered a web-based survey to nulliparous U.S.-born individuals carrying a singleton gestation of at least 28 weeks. We assessed awareness of the maternal health benefits of lactation using 10 items to create a summative score. We examined variation in awareness of these benefits by demographic characteristics, health insurance, and personal or family health history and used multivariable models to estimate associations between awareness of the maternal health benefits of lactation and breastfeeding intentions.

**Results:**

Of the 675 individuals invited to complete surveys, 451 (67%) responded. Only 50% were aware that breast-feeding lowers maternal risk of breast cancer; fewer were aware that breastfeeding lowers the risk of ovarian cancer (35%), diabetes (27%), and hypertension and heart disease (26%). Awareness of the maternal benefits of lactation did not vary by age or race/ethnicity. However, significant regional variation was noted. In multivariable models, scores of awareness of the maternal health benefits of breastfeeding were significantly associated with intentions to breastfeed for at least 12 months (adjusted odds ratio, 1.23; 95% confidence interval, 1.11, 1.37).

**Conclusions:**

Efforts to increase awareness of the maternal health benefits of lactation are still needed. Increasing awareness of the maternal health benefits of lactation may strengthen intentions to breastfeed as recommended.

In addition to the health benefits of breastmilk for children, a growing body of literature has demonstrated that lactation has important effects on the health of mothers (Chowdhury et al., 2015). Breastfeeding improves maternal–infant bonding through the psychophysiologic effects of oxytocin (Szymanska et al., 2017). Mothers who are not able to breastfeed are more likely to develop postpartum depression (Alimi et al., 2021; Howard et al., 2022). When lactation is disrupted, maternal physical health also suffers in multiple ways (Chowdhury et al., 2015). Mothers who do not breastfeed are more likely to develop breast cancer (Goldbohm & Voeding, 2002; Sung et al., 2016), ovarian cancer (Babic et al., 2020; Chowdhury et al., 2015; Luan et al., 2013), and endometrial cancer (Jordan et al., 2017).

Premature discontinuation of lactation has also been linked to maternal risk of cardiovascular disease (Nguyen et al., 2017) by impacting rates of hypertension (Stuebe et al., 2011), hyperlipidemia (Schwarz et al., 2009), and type 2 diabetes (Schwarz et al., 2010; Stuebe et al., 2005). More specifically, breastfeeding for 10 or more months has been associated with improved insulin sensitivity and glucose tolerance (Chouinard-Castonguay et al., 2013). Breastfeeding for 2 or more years is associated with lower rates of mortality from cardiovascular disease (Natland Fagerhaug et al., 2013).

Given the health benefits of breastfeeding, the U.S. Centers for Disease Control and Prevention (CDC), the American Academy of Pediatrics, the American College of Obstetricians and Gynecologists, and the World Health Organization (WHO) all recommend that mothers exclusively breastfeed for the first 6 months of their infant’s life and continue breastfeeding for at least 1 (Louis-Jacques et al., 2021; McGuire, 2014) or 2 years after birth (Meek & Noble, 2022; World Health Organization, 2021). However, only 84% of U.S. mothers initiate breastfeeding, 58% breastfeed for 6 months, and only 26% exclusively breastfeed for 6 months (CDC, 2022a). It is estimated that suboptimal breast-feeding results in 3,340 preventable U.S. deaths each year, the large majority of which are maternal (Bartick et al., 2013).

Parental awareness of the benefits of breastfeeding may impact infant feeding decisions (Ross-Cowdery et al., 2017). In prior work, maternal knowledge that breastfeeding reduces risks of breast cancer was associated with breastfeeding exclusivity and duration (Hoyt-Austin et al., 2020). Although multiple prior studies (Gorbunova et al., 2023; Patnode et al., 2016) have examined educational interventions to support breastfeeding, few interventions have focused on educating birthing parents about the effects of lactation on maternal cardiovascular health (Skouteris et al., 2017) or the role of breastfeeding in reducing rates of maternal cancers (Fan et al., 2023). As rates of breast-feeding in the United States remain suboptimal (CDC, 2022a), we sought to assess pregnant individuals’ awareness of the benefits of lactation on maternal health, to identify variables associated with awareness of these benefits, and to understand how awareness of these benefits may shape breastfeeding intentions.

## Material and Methods

We used online advertisements to recruit a convenience sample of nulliparous U.S.-born individuals carrying a singleton pregnancy of at least 28 weeks gestation. Advertisements developed for this study by StudyPages 2023 Yuzu Labs PBC were displayed on Facebook and Instagram with the goal of recruiting a diverse study population. Our sampling frame included pregnant individuals currently living in the United States who spoke English and expected their baby would live with them; we excluded those who had previously given birth, those who might not be able to breastfeed owing to HIV infection, and those with a history of breast surgery or in vitro fertilization. Each respondent provided informed consent in a fashion approved by our institutional review board. Participants completed online surveys from the location of their choice. We compensated participants for completing the online survey with a $20 gift card.

From March 2021 to February 2022, qualifying participants were invited to complete a 79-item web-based survey. We assessed awareness of the maternal health benefits of lactation using items adapted from previous work on this topic (Ross-Cowdery et al., 2017). Although the study website indicated we sought to recruit female participants expecting their first child, we did not explicitly assess participants’ gender identity. To assess participants’ awareness of the maternal health benefits of lactation, we asked a series of 10 questions; respondents first indicated (using response options of true, false, and don’t know) their awareness of whether “Breastfeeding will lower my risk of breast cancer”; similar questions were then posed regarding ovarian cancer, diabetes, high blood pressure, and heart disease. In addition, we asked respondents how breastfeeding may affect return of menses, risk of breast infections, weight retention, maternal risk of depression, arthritis, and household budgets, as detailed in the Appendix. When analyzing items assessing the maternal benefits of breastfeeding, we dichotomized responses by combining those who stated they did not know with those who provided an incorrect response. We created a 10-point summary score of awareness of the maternal benefits of breastfeeding, by giving 1 point for every correct answer provided to these questions.

We asked respondents about their intentions to breastfeed in four ways. Participants used Likert scales to indicate how much they agreed or disagreed with the following statements: “I want to breastfeed my baby” (7-point scale), “It is likely that I will try to breastfeed my baby” (7-point scale), and “Breastfeeding my baby is important” (5-point scale). We also asked how many months respondents planned to breastfeed or pump milk for their baby. We dichotomized both the 5-point and 7-point Likert scales, by comparing the top two response categories (i.e., those who strongly agreed or agreed) with the other response options (i.e., those who only somewhat agreed, neither agreed nor disagreed, or disagreed). We dichotomized data regarding how many months participants planned to breastfeed by comparing those planning to breastfeed for 12 or more months to those planning to breastfeed for less than 12 months or not at all. We examined overlap and collinearity of measures of attitudes toward breastfeeding, and then further examined the characteristics of participants who wanted to breastfeed and those who intended to breastfeed for 12 or more months.

We assessed participants’ sociodemographic characteristics using multiple items. Participants were asked, “Which of these categories best describes your racial/ethnic/cultural background?” Multiple selections were allowed from the following response options: Mixed Race, African American/Black, Arab/Middle Eastern, Asian, Hispanic/Latina, Native American/Alaskan/Pacific Islander, white/Caucasian, and other (please specify). In our analysis, we grouped all those who selected multiple responses with those who identified as mixed race, and grouped the small number of participants selecting Arab/Middle Eastern and Native American/Alaskan/Pacific Islander with those responding other. We asked participants which U.S. state they lived in, and categorized these responses into four geographic regions for analysis: Northeast (Connecticut, Maine, Massachusetts, New Hampshire, New Jersey, New York, Pennsylvania, Rhode Island, Vermont), Midwest (Illinois, Indiana, Iowa, Kansas, Michigan, Minnesota, Missouri, Nebraska, North Dakota, Ohio, South Dakota, Wisconsin), South (Alabama, Arkansas, Delaware, Florida, Georgia, Kentucky, Louisiana, Maryland, Mississippi, North Carolina, Oklahoma, South Carolina, Tennessee, Texas, Virginia, Washington, D.C., and West Virginia), and West (Alaska, Arizona, California, Colorado, Hawaii, Idaho, Montana, Nevada, New Mexico, Oregon, Utah, Washington, and Wyoming). We asked participants, “What is the highest level of school you have completed?”, with response options of “8th grade or less,” “Some high school, but did not graduate,” “High school graduate or GED,” “Some college or 2-year degree,” and “College degree (Bachelor’s or Graduate).” For this analysis, we categorized education into high school education or less, some college or 2-year degree, and college degree. We asked participants, “What type of health insurance do you have?”, with responses options of None, Private insurance, Medicaid/Medi-Cal, Military insurance/Tri-care, VA insurance, I don’t know, or other (please specify). For this analysis, we recategorized these responses as private insurance, Medicaid, or other. We assessed participants’ personal health history by asking participants, “Before you got pregnant, did you have any of the following health conditions (please select all that apply)? Diabetes (high blood sugar), hypertension (high blood pressure), asthma, depression/anxiety, or another chronic condition (please specify). Participants were also asked “During your most recent pregnancy, were you diagnosed with any of the following? Gestational diabetes, gestational hypertension, preeclampsia or eclampsia.” We assessed participant family history by asking, “Does anyone in your family have any of the following health conditions? (Please select all that apply)”: diabetes (high blood sugar), hypertension (high blood pressure), heart disease, lung disease/asthma, depression/anxiety, or breast cancer.

We used descriptive statistics to summarize sociodemographic characteristics, health-related behaviors, and awareness of the maternal health benefits of lactation. We used χ^2^ tests to determine whether awareness of the maternal health benefits of breastfeeding varied by age, race/ethnicity, geographic region, education, health insurance, and personal or family health history. We then used multivariable linear regression to determine which variables remained independently associated with breastfeeding awareness scores, when controlling for variables associated with breastfeeding in prior studies (Oberfichtner et al., 2023; Sharma et al., 2023), including maternal age, geographic region of residence, race/ethnicity, education, health insurance, personal health history, and family history. We also used multivariable logistic regression to determine whether awareness of the maternal health benefits of lactation remained independently associated with (a) wanting to breastfeed and (b) intending to breastfeed for 12 or more months, after controlling for age, education, geographic region of residence, race/ethnicity, health insurance, personal health history, and family history. Statistical significance was defined as *p* value of less than .05. All statistical analyses were conducting using SAS Enterprise Guide, Version 7.1. (Copyright 2017 SAS Institute Inc., Cary, NC).

## Results

Advertisements for this study were clicked on 3,441 times; 760 individuals expressed interest in participating and completed the screening survey. Of the 675 individuals eligible and invited to complete surveys, we obtained 451 responses (67%). We did not find significant differences between our 451 respondents and the 224 individuals who did not respond to our invitation to complete a survey in terms of race/ethnicity, age, or region of residence. The average age of respondents was 32 ± 5 years. Respondents were racially diverse: 20.2% were African American/Black, 5.5% were Asian, 4.7% were Hispanic or Latina, and 59.2% were white. Most participants (81%) were college educated; 13% were students (Table 1). Before becoming pregnant, participants reported a mean body mass index of 26 ± 6. More than one-third of participants reported a personal history of depression; additional information on respondents’ health history is shown in Table 2. Relatively few participants had been diagnosed with gestational diabetes (3.8%) or gestational hypertension (1.3%). A family history of diabetes (22.6%), hypertension (33.9%), heart disease (16.9%), or breast cancer (12.0%) was more common.

Awareness that breastfeeding lowers maternal risk of breast cancer was relatively low (50.3%). Fewer participants were aware that breastfeeding decreases the risk of ovarian cancer (35.0%), diabetes (27.1%), hypertension and heart disease (25.7%), or rheumatoid arthritis (3.3%). One-half (50.3%) were aware that breastfeeding delays return of menses and does not increase maternal risk of depression (51.0%). Fewer were aware that breastfeeding may increase risk of breast infection (26.2%). However, most (80.5%) were aware that breastfeeding would not “make it harder to ‘get my body back’ after my baby is born” and most participants (89.6%) believed breastfeeding will save their family money.

Overall, most participants felt that breastfeeding their baby was important (63.0% strongly agreed, 28.1% somewhat agreed) and almost all wanted to breastfeed their baby (83.5% strongly agreed, 8.9% agreed, and 3.1% somewhat agreed). Most indicated it was likely that they would try to breastfeed their baby (87.8% strongly agreed, 7.8% agreed, and 1.1% somewhat agreed). However, only 50.1% of participants intended to breastfeed as recommended for at least 12 months, and 9 respondents (2.3%) did not intend to breastfeed at all. There was significant collinearity between wanting to breastfeed and intending to breastfeed for 12 or more months. However, only 53% of those who strongly agreed or agreed that they wanted to breastfeed intended to breastfeed for 12 or more months, and 17% of those who did not agree or strongly agree that they wanted to breastfeed intended to breastfeed for 12 or more months.

Respondents with a greater awareness of the maternal health benefits of breastfeeding were more likely to agree or strongly agree that they wanted to breastfeed. Among those who agreed or strongly agreed they wanted to breastfeed, scores of awareness of the maternal benefits of breastfeeding averaged 4.5 compared with 3.1 among those who only somewhat or did not want to breastfeed (*p* = .003). Those who agreed or strongly agreed they wanted to breastfeed were significantly more likely to be aware that breastfeeding would not make it harder to “get my body back,” or increase risk of depression, and would delay return of menses, reduce risk of breast cancer, and reduce risk of ovarian cancer (Table 3). Similarly, awareness of the maternal benefits of breastfeeding was greater among respondents who did vs. did not plan to breastfeed for at least 1 year (average awareness score of 4.8 vs. 3.9; *p* < .001). Awareness of the maternal benefits of breastfeeding were lowest among the nine respondents who did not plan to breastfeed at all, average scores of 2.8 ± 1.9.

We found no significant differences in awareness of the maternal benefits of breastfeeding by age, race/ethnicity, or family health history. In bivariate analyses (Table 4), awareness of the maternal health benefits among participants who had Medicaid insurance was lower than among those who were privately insured (*p* = .03); those who had college degrees had significantly higher awareness scores (*p* = .0004) than those who were less educated. In bivariate and multivariable analyses (Table 4), participants who lived in the Midwest were significantly more likely than those who lived in the Western United States to be aware of the maternal health benefits of lactation (*p* = .04), and those who reported a history of depression or anxiety were significantly less likely to be aware of the maternal health benefits of lactation (*p* = .0006). In multivariable models (Table 5), accounting for sociodemographic characteristics and participants’ personal health history, awareness of the maternal health benefits of breastfeeding remained associated with both wanting to breastfeed and intending to breastfeed for at least 12 months.

## Discussion

In this sample of U.S. individuals pregnant with their first child, we found that most respondents remained unaware of the maternal health benefits of breastfeeding. This finding is particularly notable because these respondents were highly educated: 81% were college educated, compared with 39% of U.S. women overall (Duffin, 2022). Further, respondents were older than the average first-time mother in the United States (32 years vs. 26 years) (CDC, 2016). Although awareness of the benefits of lactation in reducing risk of breast cancer was greater in this highly educated sample than previously reported among the larger U.S. population (Hoyt-Austin et al., 2020), only one-half were aware of this potentially modifiable risk factor for breast cancer, which afflicts one in eight U.S. women across their life course (CDC, 2022b). Similarly, cardiovascular disease is the leading cause of death among U.S. women (SEER Cancer Statistics Review, 1975–2017, n.d.), and few participants were aware that breastfeeding may lower the risk of hypertension (Stuebe et al., 2011) and improve cardiovascular health in later life (Nguyen et al., 2017). Of concern, participants with a prior diagnosis of hypertension were less likely to report they strongly wanted to breastfeed, indicating prenatal counseling on the benefits of breastfeeding may be particularly important for pregnant individuals with hypertension.

Our findings are in line with prior studies that have also found that U.S. women have limited awareness of the maternal health benefits of lactation (Hoyt-Austin et al., 2020; Ross-Cowdery et al., 2017). Our finding that relatively small differences in awareness scores were significantly associated with intentions to breastfeed as recommended for at least 1 year highlights the importance of addressing the maternal health benefits of lactation when providing prenatal counseling. Prior studies have also found that mothers with greater awareness of the maternal benefits of breastfeeding were more likely to express a desire to breastfeed (Ross-Cowdery et al., 2017). Because mothers with strong prenatal intentions to breastfeed most frequently breastfeed as recommended (Bai et al., 2010), prenatal counseling about the maternal health benefits of lactation has the potential to meaningfully impact infant feeding behaviors and improve health outcomes across two generations. Notably in this study, awareness of the maternal benefits of breastfeeding did not vary by maternal race/ethnicity; however, intention to breastfeed for 12 or more months was lower among Black participants, those of mixed race, and those of races other than Black, Asian, Hispanic or Latina, or white, compared with non-Hispanic white participants. This finding highlights the need for additional targeted interventions to address breastfeeding inequities among these groups (Chiang et al., 2021; Rabb et al., 2023).

Limitations of this study include the understanding that observed associations may not reflect causal relationships and that social desirability bias may have impacted reported intentions to breastfeed. Further, surveys were only distributed in English and the population studied is more educated than the larger U.S. population. Intentions to breastfeed and breastfeeding rates tend to be higher among more educated U.S. women (Lee et al., 2005); therefore, awareness of the maternal benefits of breastfeeding is likely even lower among U.S. women who are less educated than those we studied. Because this study is not a population-based sample, the results cannot be generalized to the U.S. population; the study participants’ average age of 32 years is older than the national average of 30 years for first-time mothers in the United States (Morse, 2022). We did not measure other factors that can influence breastfeeding intentions, such as social or cultural norms, partner and family influence, and media representations of breastfeeding (Matriano, 2022). Additionally, awareness regarding whether breastfeeding will “save my family money” is complex and reflects the individual’s perception of the time required to breastfeed or express milk, their earning potential, as well as access to programs such as the Special Supplemental Nutrition Program for Women, Infants, and Children, which subsidize the cost of alternatives to breast milk. We recognize that not all pregnant individuals identify as female, women, or moms, which were the terms used when recruiting participants for this study; further research is needed to understand how to best support informed decisions regarding infant feeding among pregnant individuals who do not identify as women (Gribble et al., 2023; Yang et al., 2023).

## Implications for Policy and/or Practice

Efforts are still needed to increase awareness of the maternal health benefits of lactation alongside other initiatives to increase breastfeeding rates. More specifically, these findings indicate a need for prenatal counseling about the maternal benefits of breastfeeding and tailored lactation support for high-risk groups, such as women who identify as Black or have a history of hypertension. Recognizing the complex nature of infant feeding decisions, efforts to increase awareness of the maternal benefits of breastfeeding must be combined with advocacy for paid parental leave, baby-friendly maternity care practices, and expanding access to prompt postpartum lactation consultation (Huang et al., 2019); without such supports, even those who would like to breastfeed often find it hard to do so. Future research should examine how gender identity impacts awareness of the maternal benefits of breastfeeding.

## Conclusions

Although multiple factors influence infant feeding decisions, low levels of awareness of the maternal benefits of breastfeeding, even among a highly educated sample of pregnant individuals, indicate that prenatal educational efforts remain needed to ensure that U.S. families appreciate the long-term effects of breastfeeding on the health of both birthing parents and their children.

## Supplementary Material

Supplementary material

## Figures and Tables

**Table 1:** Demographic Characteristics of Responding Nulliparous U.S. Pregnant Individuals (*N* = 451*)

Demographic Characteristic	No.	%
Age, years	31.6 ± 5.0	
Race/Ethnicity		
African American/Black	91	20.2
Asian	25	5.5
Hispanic or Latina	21	4.7
Mixed race	33	7.3
Native American, Alaskan, other	7	1.6
White	267	59.2
Geographic region of residence^†^		
Northeast	68	15.1
Midwest	81	18.0
South	192	42.6
West	87	19.3
Education		
College degree (bachelor’s or graduate)	364	80.7
Some college or 2-year degree	59	13.1
High school or less	26	5.8
Health insurance		
Private	326	72.3
Medicaid	96	21.3
Other^‡^	29	6.4

* Data were missing on age for 19, race/ethnicity for 7, geographic region for 23, education for 2, and health insurance for 0 participants.

† Geographic regions defined as follows: Northeast (Connecticut, Maine, Massachusetts, New Hampshire, New Jersey, New York, Pennsylvania, Rhode Island, Vermont), Midwest (Illinois, Indiana, Iowa, Kansas, Michigan, Minnesota, Missouri, Nebraska, North Dakota, Ohio, South Dakota, Wisconsin), South (Alabama, Arkansas, Delaware, Florida, Georgia, Kentucky, Louisiana, Maryland, Mississippi, North Carolina, Oklahoma, South Carolina, Tennessee, Texas, Virginia, Washington, D.C., and West Virginia), and West (Alaska, Arizona, California, Colorado, Hawaii, Idaho, Montana, Nevada, New Mexico, Oregon, Utah, Washington, and Wyoming).

‡ Other includes answer responses of “Military Insurance,” “Other,” and “I Don’t Know.”

**Table 2:** Personal and Family Health History of Responding U.S. Pregnant Individuals (*N* = 451)

Health Characteristic	No.	%
Personal medical history		
Depression	150	33.6
Hypertension	6	1.3
Diabetes	4	0.9
Other chronic conditions	53	11.8
Gestational diabetes	17	3.8
Gestational hypertension or pre-eclampsia	6	1.3
BMI before pregnancy		
Underweight (BMI<18.5)	14	3.1
Healthy (BMI 18.5 to <25)	241	53.4
Overweight (BMI 25 to <30)	108	24.0
Obese (BMI ≥30)	86	19.1
Family medical history		
Depression	200	44.4
Diabetes	102	22.6
Lung disease	85	18.9
Heart disease	76	16.9
Breast cancer	54	12.0
Hypertension	153	33.9

*Abbreviation:* BMI, body mass index.

**Table 3:** Awareness of the Maternal Benefits of Lactation Among Nulliparous Pregnant Individuals by Attitudes Toward Breastfeeding and Intended Duration of Breastfeeding

Breastfeeding Will…	Total (*N* = 451)	Wants to Breastfeed			Intended Duration of Breastfeeding		
		Strongly Agree* (*n* = 415)	Less Strongly (*n* = 36)	*p* Value	<12 Months (*n* = 223)	≥12 Months (*n* = 228)	*p* Value
Not make it hard to get my body back	80.5	82.7	55.6	<.001	79.5	81.6	.56
Delay return of menses	50.3	52.1	30.6	.01	43.1	57.5	.002
Reduce risk of breast cancer	50.3	51.8	33.3	.03	44.5	50.6	.01
Not increase risk of depression	51.2	52.5	36.1	.06	46.2	56.1	.03
Reduce risk of ovarian cancer	35.0	36.9	13.9	.006	29.2	40.8	.0096
Reduce risk of diabetes	27.1	27.5	22.2	.50	21.5	32.5	.009
Reduce risk of high blood pressure and heart disease	25.7	26.3	19.4	.37	20.6	30.7	.01
Increase risk of breast infections	25.8	25.8	25.0	.92	24.2	27.2	.47
Reduce risk of arthritis	3.3	3.4	2.8	.84	2.7	4.0	.46
Save my family money	89.6	90.8	75.0	.003	83.0	96.1	<.001

Note: Values are percent unless otherwise noted.

* Dichotomized responses by comparing those who strongly agreed or agreed to those who only somewhat agreed, neither agreed nor disagreed, or disagreed.

**Table 4:** Variables Associated With Awareness of the Maternal Benefits of Breastfeeding Among Responding U.S. Pregnant Individuals (*N* = 451)

Independent Variable	Awareness of Maternal Benefits, Average Score*	Bivariate Model Estimate (95% CI)	Multivariable Model^†^ Estimate (95% CI)
Age			
<30 years	4.35 ± 2.3	−0.10 (−0.56, 0.37)	0.10 (-0.41, 0.61)
≥30 years	4.45 ± 2.10	reference	reference
Race/ethnicity			
African American/Black	4.3 ± 2.3	−0.28 (−0.80, 0.24)	−0.19 (−0.79, 0.40)
Asian	4.3 ± 2.4	−0.21 (−1.10, 0.68)	−0.47 (−1.42, 0.47)
Hispanic or Latina	4.2 ± 2.3	−0.34 (−1.31, 0.63)	−0.02 (−0.98, 1.01)
Mixed race, Native American, Alaskan, other	3.75 ± 2.10	**−0.78 (−1.50, −0.06)**	−0.70 (-1.50, 0.12)
White	4.5 ± 2.1	reference	reference
Geographic region of residence^‡^			
West	4.0 ± 2.0	reference	Reference
Midwest	4.7 ± 2.2	**0.68 (0.03, 1.33)**	**0.78 (0.09, 1.46)**
South	4.3 ± 2.2	0.25 (−0.29, 0.79)	0.37 (−0.21, 0.95)
Northeast	4.5 ± 2.1	0.44 (−0.24, 1.12)	0.39 (−0.33, 1.11)
Education			
College degree (bachelor’s or graduate)	**4.6 ± 2.1**	**0.91 (0.40, 1.41)**	0.55 (−0.11, 1.21)
No college degree	3.7 ± 2.3	reference	Reference
Health insurance			
Private	4.5 ± 2.1	reference	Reference
Medicaid	3.9 ± 2.4	**−0.58 (−1.08, −0.09)**	−0.13 (-0.76, 0.51)
Other^§^	4.9 ± 2.5	0.37 (−0.45, 1.20)	0.26 (−0.64, 1.16)
Personal health history			
Obesity	4.2 ± 2.2	−0.26 (−0.25, 0.78)	0.15 (−0.42, 0.72)
Diabetes	3.0 ± 0.8	−1.40 (−3.55, 0.75)	−1.30 (−3.42, 0.83)
Hypertension	4.5 ± 3.2	0.11 (−1.65, 1.87)	0.79 (−0.96, 2.54)
Depression/anxiety	3.9 ± 2.2	**−0.74 (−1.16, −0.32)**	**−0.67 (−1.13, −0.22)**
Family history			
Breast cancer	4.7 ± 2.1	0.38 (−0.24, 1.00)	0.39 (−0.25, 1.02)
Diabetes	4.4 ± 2.0	−0.11 (−0.59, 0.37)	0.05 (−0.49, 0.58)
Heart disease	4.6 ± 2.3	0.29 (−0.27, 0.83)	0.14 (−0.43, 0.70)

*Abbreviation:* CI, confidence interval.

Note: Bold font indicates relationships that are significant (*p* < .05).

* Awareness scores (ranging from 0 to 10) were calculated by summing the responses to 10 questions regarding the maternal health effects of lactation detailed in the Appendix.

† Multivariable model includes all variables shown in table, including age, race/ethnicity, geographic region of residence, education, health insurance, personal health history, and family history.

‡ Geographic regions defined as follows: Northeast (Connecticut, Maine, Massachusetts, New Hampshire, New Jersey, New York, Pennsylvania, Rhode Island, Vermont), Midwest (Illinois, Indiana, Iowa, Kansas, Michigan, Minnesota, Missouri, Nebraska, North Dakota, Ohio, South Dakota, Wisconsin), South (Alabama, Arkansas, Delaware, Florida, Georgia, Kentucky, Louisiana, Maryland, Mississippi, North Carolina, Oklahoma, South Carolina, Tennessee, Texas, Virginia, Washington, D.C., and West Virginia), and West (Alaska, Arizona, California, Colorado, Hawaii, Idaho, Montana, Nevada, New Mexico, Oregon, Utah, Washington, and Wyoming).

§ Other includes answer responses of “Military Insurance,” “Other,” and “I Don’t Know.”

**Table 5:** Awareness of the Maternal Benefits of Breastfeeding, Attitudes Toward Breastfeeding, and Intentions to Breastfeed Among Nulliparous Pregnant Individuals in the United States

Independent Variable	Strongly Agreed They Want to Breastfeed*		Intended Breastfeeding Duration of ≥12 Months	
	Bivariate OR (95% Cl)	Multivariate^†^ OR (95% Cl)	Bivariate OR (95% CI)	Multivariate^†^ OR (95% CI)
Breastfeeding Awareness Score	**1.38 (1.15, 1.65)**	**1.32 (1.08, 1.62)**	**1.21 (1.10, 1.33)**	**1.23 (1.11, 1.37)**
Age				
<30 years	0.04 (−0.02, 0.10)	2.58 (0.88, 7.61)	1.03 (0.67, 1.58)	1.18 (0.71, 1.97)
≥30 years	reference	reference	reference	Reference
Race				
Asian	0.53 (0.15, 1.94)	0.53 (0.13, 2.14)	0.89 (0.39, 2.04)	0.90 (0.36, 2.28)
Black	0.66 (0.29, 1.94)	1.37 (0.48, 3.91)	**0.40 (0.24, 0.65)**	**0.39 (0.21, 0.73)**
Hispanic or Latina	1.45 (0.18, 11.40)	1.62 (0.19, 13.77)	0.53 (0.21, 1.29)	0.41 (0.15, 1.12)
Mixed race or other	0.89 (0.25, 3.18)	2.20 (0.45, 10.71)	**0.37 (0.19, 0.76)**	0.49 (0.21, 1.14)
White	reference	reference	reference	Reference
Geographic region of residence^‡^				
West	reference	reference	reference	Reference
Midwest	1.59 (0.37, 6.86)	1.33 (0.28, 6.33)	**0.44 (0.23, 0.82)**	**0.38 (0.19, 0.78)**
South	0.50 (0.18, 1.36)	0.48 (0.16, 1.44)	**0.37 (0.21, 0.62)**	**0.41 (0.23, 0.75)**
Northeast	0.77 (0.21, 2.77)	0.73 (0.17, 3.14)	**0.33 (0.17, 0.65)**	**0.36 (0.17, 0.75)**
Health insurance				
Private	reference	reference	reference	Reference
Medicaid	0.48 (0.23, 1.02)	0.73 (0.17, 3.14)	0.70 (0.44, 1.11)	0.84 (0.44, 1.61)
Other^∥^	0.60 (0.17, 2.13)	1.44 (0.18, 11.85)	1.79 (0.81, 3.96)	**2.80 (1.05, 7.42)**
Education				
College degree^§^	**2.61 (1.26, 5.38)**	**1.01 (1.01, 9.28)**	1.60 (0.98, 2.6)	1.21 (0.62, 2.38)
No college degree	reference	reference	reference	Reference
Personal health history				
Obesity	1.54 (0.57, 3.99)	1.78 (0.58, 5.44)	0.97 (0.67, 1.55)	1.73 (0.97, 3.07)
Hypertension	**0.17 (0.03, 0.94)**	0.14 (0.02, 1.11)	1.97 (0.36, 10.9)	2.76 (0.41, 18.62)
Depression/anxiety	0.77 (0.38, 1.54)	0.97 (0.41, 2.30)	1.9 (0.73, 1.61)	1.15 (0.72, 1.82)

*Abbreviations:* CI, confidence interval, OR, odds ratio.

Note: Bold font indicates relationships that are significant (*p* < .05).

* Respondents indicating “strongly agree” or “agree” vs. all other responses on a 7-point scale when asked how much they agreed with the statement “I want to breastfeed my baby.”

† Multivariate models adjusted for all variables shown in table.

‡ Geographic regions defined as follows: Northeast (Connecticut, Maine, Massachusetts, New Hampshire, New Jersey, New York, Pennsylvania, Rhode Island, Vermont), Midwest (Illinois, Indiana, Iowa, Kansas, Michigan, Minnesota, Missouri, Nebraska, North Dakota, Ohio, South Dakota, Wisconsin), South (Alabama, Arkansas, Delaware, Florida, Georgia, Kentucky, Louisiana, Maryland, Mississippi, North Carolina, Oklahoma, South Carolina, Tennessee, Texas, Virginia, Washington, D.C., and West Virginia), and West (Alaska, Arizona, California, Colorado, Hawaii, Idaho, Montana, Nevada, New Mexico, Oregon, Utah, Washington, and Wyoming).

§ Bachelor’s or graduate degree.

∥ Other includes responses of “Military Insurance,” “Other,” and “I Don’t Know.”
