# Supplementary material for: Awareness of the Maternal Health Benefits of Lactation Among U.S. Pregnant Individuals
Source: Womens Health Issues. Author manuscript; Available in PMC 2025 May 28. (PMC12118535; doi:10.1016/j.whi.2023.12.004)
Supplement: Supplementary material [file NIHMS2082474-supplement-Supplementary_material.docx]

**For each of the following statements, please choose the answer that best describes your opinion of whether breastfeeding affects mothers' health in the following ways.**

| **Breastfeeding will...** | True | False | I don't know | Coded as Correct |
| --- | --- | --- | --- | --- |
| Lower my risk of breast cancer |  |  |  | True |
| Lower my risk of ovarian cancer |  |  |  | True |
| Lower my risk of developing diabetes |  |  |  | True |
| Increase my risk of a breast infection |  |  |  | True |
| Increase my risk of depression |  |  |  | False |
| Make it harder to "get my body back" after my baby is born |  |  |  | False |
| Protect my hands from arthritis |  |  |  | True |
| Lower my blood pressure and risk of heart disease |  |  |  | True |
| Delay my menstrual period after my baby is born |  |  |  | True |
| Save my family money |  |  |  | True |
